# Supplementary material for: Detailed molecular and epigenetic characterization of the pig IPEC-J2 and chicken SL-29 cell lines
Source: iScience. 2023 Feb 20;26(3):106252. doi: 10.1016/j.isci.2023.106252 (PMC10018572; doi:10.1016/j.isci.2023.106252)
Supplement: Data S2. Complete homer output for identified motifs in Chicken SL-29, related to Tables 5 and 6 — Homer motif analysis results for histone modifications H3K4me1, H3K4me3, H3K27ac, enhancers, and ATAC-seq of chicken SL-29 cell line. Parameters for possible false positives is as mentioned earlier for S5. [file mmc3.zip › Data_S2/S6/Chicken_SL_29/peak_files_H3K4me3/homerResults/motif6.similar.html]

motif6

## Information for motif6

T
C
A
G
C
T
A
G
A
C
T
G
A
T
G
C
G
A
T
C
A
G
C
T
T
A
C
G
T
C
G
A
T
A
C
G
A
T
C
G
  
Reverse Opposite:  

T
A
G
C
A
T
G
C
A
G
C
T
A
T
G
C
T
C
G
A
C
T
A
G
T
A
C
G
T
G
A
C
G
A
T
C
A
G
T
C
  

|  |  |
| --- | --- |
| p-value: | 1e-63 |
| log p-value: | -1.469e+02 |
| Information Content per bp: | 1.459 |
| Number of Target Sequences with motif | 4185.0 |
| Percentage of Target Sequences with motif | 24.38% |
| Number of Background Sequences with motif | 6239.1 |
| Percentage of Background Sequences with motif | 19.13% |
| Average Position of motif in Targets | 150.1 +/- 78.7bp |
| Average Position of motif in Background | 149.6 +/- 95.8bp |
| Strand Bias (log2 ratio + to - strand density) | 0.0 |
| Multiplicity (# of sites on avg that occur together) | 1.73 |
| Motif File: | file (matrix) reverse opposite |

### Similar de novo motifs found

|  |  |  |  |  |  |  |  |
| --- | --- | --- | --- | --- | --- | --- | --- |
| Rank | Match Score | Redundant Motif | P-value | log P-value | % of Targets | % of Background | Motif file |
| 1 | 0.725 | T A G C C G T A C A T G C T A G A T G C T A G C A T G C T A C G | 1e-52 | -121.634363 | 38.02% | 32.45% | motif file (matrix) |
| 2 | 0.847 | A G T C A T C G C T G A A C T G A C T G A G T C A G T C A G T C | 1e-49 | -113.578932 | 29.00% | 24.06% | motif file (matrix) |
| 3 | 0.630 | T A C G T A C G A T C G A T G C A G T C A G C T A T C G A C T G A T C G T A G C A G T C A C G T A T C G | 1e-29 | -68.425347 | 4.51% | 2.93% | motif file (matrix) |
| 4 | 0.607 | A C G T A C T G A C G T C T A G A C T G A C T G A C T G T G A C A G T C A T G C | 1e-29 | -67.695077 | 2.04% | 1.05% | motif file (matrix) |
| 5 | 0.648 | T C G A T A C G T A C G A T G C T A G C A T C G A T C G A T C G A T G C A T G C A T G C A T C G A C G T | 1e-16 | -37.653079 | 4.62% | 3.41% | motif file (matrix) |
